# Governance of dietary microplastic exposure risk in marine ranching in the context of climate change

**DOI:** 10.3389/fpubh.2026.1975500

**Published:** 2026-10-06

**Authors:** Shan Zheng, Lianghong Yu, Suowen Li, Xiaolei Liu

**Affiliations:** 1 School of International Business, Qingdao Huanghai University, Qingdao, China; 2 School of Advanced Agricultural Sciences, Peking University, Beijing, China

**Keywords:** climate change, collaborative governance, dietary exposure, evolutionary game, marine ranching, microplastics

## Abstract

Aquaculture facilities in marine ranches continuously fragment and release microplastics, which accumulate in filter-feeding shellfish and enter residents’ diets. Climate change intensifies this pathway. Warming and ultraviolet exposure accelerate the fragmentation of aquaculture plastics, while typhoons damage facilities and release microplastics in pulses and raise the health expenditure at stake for the government, enterprises, and households. This study constructs a tripartite evolutionary game model of the government, marine ranching enterprises, and consumers. Numerical simulation is used to examine how each actor’s strategies shape the system’s convergence path and speed and long-term residual exposure. The findings are as follows. Whether the government can sustain regulation depends solely on whether the process-based assessment payoff covers the cost of regulation, because the outcome-based performance payoff can be free-ridden and cancels out in the payoff differential. Once subsidies exceed this government-side ceiling, they crowd out regulation and cause exposure to rebound. Enterprises’ tolerance for plastic-reduction costs follows a tiered threshold structure, and the simultaneous phase-down of regulation and subsidies readily triggers a governance relapse, so a mature market premium mechanism must be in place first. Consumer participation is subject to a self-undermining mechanism whereby governance success erodes the incentive to participate. Sustaining it requires lowering information and oversight costs rather than heightening risk perception. Information disclosure relaxes the consumer participation constraint in all configurations without fiscal expenditure, making it the most cost-effective instrument. When none of the equilibrium conditions holds, the system oscillates periodically, and population-average exposure underestimates both the cumulative health cost and the risk borne by highly exposed subgroups. The results lock in the government’s presence through process-based assessment, cultivate market premiums before phasing down regulation, and integrate microplastic source control into climate adaptation planning for marine ranching.

## 1 Introduction

Plastic pollution is evolving from an environmental issue into a public health issue, and climate change is tightening the coupling between the two. The two crises are now recognized as fundamentally linked rather than separate. This is because the plastic life cycle is a growing source of greenhouse gas emissions while the more frequent extreme weather and floods associated with climate change exacerbate the dispersal of plastic already present in the environment (1). Roughly 8.3 billion tonnes of virgin plastics have been produced globally, of which about 60% has been discarded in landfills or the natural environment (2). There, sunlight, mechanical abrasion, and biological action continuously fragment it into microplastics smaller than 5 mm. These particles enter the human body through dietary and respiratory pathways. U. S. residents are estimated to ingest 39,000 to 52,000 particles per year through food alone (3), the median daily intakes of children and adults are 553 and 883 particles respectively, and the particles can accumulate irreversibly in human tissues (4, 5). Biomonitoring studies have successively quantified multiple polymer particles in stool (6), the placenta (7), and peripheral blood (8). A prospective observational study further found that patients in whom microplastics and nanoplastics were detected in carotid plaques. These patients faced a roughly 4.5-fold higher risk of the composite endpoint of myocardial infarction, stroke, and all-cause death than those without detectable particles (9). It provides the first population-level direct evidence linking microplastic exposure to adverse health outcomes. Substantial knowledge gaps remain regarding dose–response relationships and pathogenic mechanisms (10), and the available data are insufficient to derive definitive safety thresholds (11). Even so, interdisciplinary commission reports have already listed exposure reduction across the entire plastic life cycle as a public health priority (12). Implementing precautionary controls before the evidence is complete has become a basic consensus in the public health field.

Among all exposure pathways, dietary intake is the principal channel of population microplastic exposure, and aquatic products are among the food categories carrying the most concentrated loads. Foundational studies quantified microplastics in the soft tissues of commercially farmed blue mussels and Pacific oysters, showing that shellfish alone could raise the annual dietary exposure of European consumers to about 11,000 particles (13). A mussel survey covering 22 coastal sites in China likewise reported loads of 0.9 to 4.6 particles per gram wet weight (14). A systematic review and meta-analysis of 50 studies further confirmed that mollusks are the aquatic category with the highest microplastic content per unit mass. Per capita annual intake reaches about 55,000 particles under the highest-consumption scenario (15). Because bivalves such as oysters and mussels directly concentrate suspended particles through filter feeding and are usually eaten whole together with the digestive gland. The dedicated assessments both identified them as priority categories for dietary exposure (16). Their risk level depends heavily on the aquaculture water environment and on source control at the production end.

Marine ranching provides a typical setting for examining this risk, one in which sources and exposure are tightly coupled (17). The buoys, netting, ropes, and raft frames that support its operation make extensive use of plastics such as expanded polystyrene and polyethylene. These continuously fragment and shed under seawater scouring, ultraviolet aging, and biological erosion. The aquaculture facilities themselves become an important source of microplastics in the sea area (16, 18). Climate risk is intensifying this source. Warming and ultraviolet exposure shorten the service life of facilities and raise the rate at which they shed fragments. Climate change is also increasing the intensity of extreme weather, including tropical storms (1). They damage and disperse aquaculture gear and resuspend contaminated sediments. After two typhoons in Sanggou Bay, an intensively farmed bay on the Shandong Peninsula, the average microplastic abundance in seawater and sediments rose by roughly 40%. The particle loads of cultured oysters increased in step (19). The aquaculture-derived microplastic flux is therefore not a stationary background but a climate-sensitive hazard that can be expected to rise with climate risk. A survey of Xiangshan Bay, an intensively farmed embayment, showed that aquaculture-derived microplastics accounted for 55.7 and 36.8% of the total microplastics in surface seawater and sediments, respectively, (20). Accumulation in the water column, sediments, and farmed organisms is coupled *in situ* within the aquaculture waters (21, 22). End-of-pipe measures such as depuration can reduce microplastic content in mussels by only about 29 to 47% (23) and cannot substitute for source reduction, so the focus of governance must shift forward to the production end. The result is a short, tightly closed risk pathway. Facilities release microplastics *in situ* into the aquaculture waters, filter-feeding shellfish and demersal fish ingest and concentrate them *in situ*. The products enter residents’ diets through market circulation, and the exposure consequences are ultimately borne by consumers. Viewed through the lens of climate risk and health expenditure, this pathway adds a pollution-mediated channel to a climate-sensitive health burden that is already growing worldwide and whose economic costs fall on governments, firms, and households alike (24). Ten climate-sensitive events in the United States in 2012 alone generated an estimated US$10 billion in health-related costs, most of which were never counted in official damage assessments. In China the health burden of heatwaves and other climate hazards now runs far above its historical baseline and is concentrated in coastal cities. This is precisely where marine ranching is clustered (25). In the marine ranching setting the distribution of this burden mirrors the three-sector structure emphasized in that literature: the government bears medical security, monitoring, and emergency expenditure. Enterprises bear retrofit, rectification, and reputational costs. Consumers bear the health loss of exposure together with the cost of protective behavior, and unlike the direct damage of a heatwave or a storm, this distribution is endogenous to how the three actors behave. This misaligned structure, in which the production side generates risk while the consumption side bears it. Combined with the long latency of risk manifestation, the high technical threshold of detection, and the difficulty of source attribution, it means that no single actor can accomplish risk control independently. Government regulation is constrained by regulatory costs and detection probabilities, enterprises’ plastic-reduction retrofits are constrained by whether the incremental costs can be recovered. Consumers’ protection and oversight are constrained by the availability of risk information. China’s marine ranches face a continually expanding functional scope, with growing facility scale and industrial volume (26–28), intensifying typhoon and other climate risks along China’s coast (25), and rising public risk awareness. A key question is how to design a collaborative governance mechanism that simultaneously activates the government, enterprises, and consumers. This is an urgent question of both public health and industrial sustainability significance.

Viewed against the theoretical literature, this question sits at the intersection of microplastic risk research and multi-actor governance research. Existing microplastic research is dominated by the natural science paradigm, focusing on source apportionment, environmental occurrence, monitoring methods, bioaccumulation, and toxicological effects. It has built an exposure evidence chain from environmental media to human tissues. A systematic review of two decades of progress in the field pointed out that evidence on the effectiveness of governance measures has become the most pressing current knowledge need (29, 30). Echoing this accumulation of evidence, the regulatory agenda is also accelerating. Scholars have called for a legally binding global agreement covering the entire plastic life cycle (31) and stressed that microplastic regulation must strike a fine balance between controlling particle release and incentivizing material innovation (32). In parallel, the literature on climate risk and health expenditure has begun to quantify the fiscal and household costs of climate-sensitive events and to call for climate-resilient health financing (24). However, it has seldom examined pollution pathways whose hazard is itself amplified by climate change. Polycentric governance theory holds that the governance of complex public affairs is often beyond the capacity of either a pure market or a single government and depends instead on the sustained interaction of multiple decision centers under rule constraints. The social-ecological systems framework further shows that governance performance depends on the fit among the attributes of the resource system, the governance system, and the users (33). Marine ranches are precisely such systems, with highly clustered resource users and nested governance tiers. Methodologically, evolutionary game theory originated from the concept of the evolutionarily stable strategy and the replicator dynamic equation in biology. After being introduced into economics and the social sciences it has become the standard analytical tool for characterizing strategic interaction among boundedly rational populations (34). Premised on bounded rationality and strategy learning, it uses replicator dynamic equations to describe the evolution of strategy shares within populations. The framework has been widely applied to mechanism analysis of government–enterprise strategic interaction under carbon taxes and subsidies (35, 36). It has also been extended in the context of collaborative environmental pollution governance to a tripartite game of the government, enterprises, and the public (37, 38). Its capacity to characterize the three-way interaction among regulators, the regulated, and the affected public has been repeatedly validated. In recent years this approach has begun to enter the field of marine ranching governance. Existing studies have constructed a tripartite regulatory evolutionary game model for the ecological security of marine ranches (39). Others have incorporated consumer preferences as an exogenous parameter into the evolutionary analysis of marine ranching enterprises’ technological innovation strategies under government incentive policies (40). However, their risk objects concentrate on ecological security and industrial upgrading, and the public health dimension of dietary microplastic exposure has not yet entered their problem domain.

Examining the governance of dietary microplastic exposure in marine ranching along this thread reveals four shortcomings in the existing research. First, there is a disciplinary gap in the research object. The health risk literature has completed the construction of the environment–food–human evidence chain and pinpointed bivalve shellfish as key exposure vehicles. Yet its governance discussion remains at the level of principle-based qualitative recommendations. Governance research possesses mature formal tools but has not yet brought the marine ranching setting, where sources and exposure are tightly coupled, and its dietary exposure risk into the scope of modeling. Second, in terms of actor structure, most models adopt a government–producer dyadic framework in which consumers are either exogenized as demand parameters or, even when included, assigned only a single purchasing function. Their dual channels of pulling enterprise payoffs through green willingness to pay and raising enforcement efficacy through reporting and oversight are not characterized simultaneously, so enterprises’ compliance incentives are systematically underestimated. Third, in terms of methodological focus, existing studies generally stop at determining asymptotic stability conditions and discussing the existence of ideal equilibria. They treat parameter regions where no evolutionarily stable strategy exists as mathematical corner cases not worth developing. The convergence speed, the convergence path, and the policy and health implications carried by persistent oscillation in the absence of a stable equilibrium are rarely addressed. Fourth, in terms of mechanism characterization, although the existence of a subsidy ceiling is already a consensus in environmental regulation research. Existing explanations concentrate on the enterprise side, while the government’s own incentive and constraint for sustaining regulation has not been incorporated into models. Meanwhile, the information asymmetry induced by microplastic content as a typical credence attribute and the governance value of information disclosure also lack formal treatment.

To address these gaps, this study takes the dietary microplastic exposure risk of marine ranching, a climate-sensitive pathway of health expenditure, as its object, constructs a tripartite evolutionary game model comprising the government, marine ranching enterprises, and consumers, derives the asymptotic stability conditions of all pure-strategy equilibria through replicator dynamic equations and the Jacobian matrix method, and then uses numerical simulation to examine the system’s convergence paths and speeds under key parameters, the marginal effectiveness of policy instruments, the parameter phase diagram of governance configurations, and the robustness of the conclusions. Relative to the existing literature, the marginal contributions of this study are as follows.

First, by endogenizing consumers as an independent strategic actor, this study identifies the dual leverage effect of consumption-side participation on enterprise plastic reduction. Active participation simultaneously amplifies enterprises’ net compliance payoff through the full green premium and the raised detection probability. This study further reveals a self-undermining mechanism whereby governance success erodes participation incentives. The more successful source reduction is, the lower the marginal health benefit of consumption-side protection, and the harder it is for participation to sustain itself spontaneously. Second, this study identifies the periodic oscillation that arises when no evolutionarily stable strategy exists as an independent class of dietary exposure scenario rather than a transition between steady states. It points out that evaluating the oscillatory state by population-average exposure simultaneously underestimates the cumulative health cost and the risk of highly exposed subgroups. This adds a previously missing scenario dimension to dietary exposure risk assessment from the perspective of governance dynamics. Third, this study proves that information disclosure unilaterally relaxes consumers’ participation constraint in all strategy configurations without requiring additional fiscal expenditure. It thereby provides a mechanism-based rationale, internal to the model, for ranking information disclosure ahead of marginal adjustments to subsidies and fines. Taken together, the results provide a mechanism-based account of how the health expenditure generated by a climate-sensitive pollution pathway is distributed among the government, enterprises, and households. It also identifies which policy instruments can shift that distribution at the lowest fiscal cost.

## 2 Materials and methods

### 2.1 Real-world context and the collaborative governance framework

The governance of microplastics in marine ranching faces three real-world predicaments. The first is information asymmetry. The microplastic load of aquatic products can be measured only through standardized procedures of digestion, density separation, microscopic examination, and polymer spectroscopic identification. Strict quality control requirements apply for blank controls and contamination prevention (41). Consumers cannot verify it through the senses or consumption experience either before or after purchase, so microplastic content is a typical credence attribute (42). In the absence of authoritative test disclosure and unified labeling, the market cannot spontaneously form a quality premium that distinguishes superior from inferior products, and plastic-reduced products instead face adverse selection pressure. The second is incomplete enforcement. Microplastics in the sea must be attributed among multiple pathways: land-based runoff, shipping and port activities, and the fragmentation of aquaculture facilities, so evidence collection is difficult and slow and violations are hard to verify. The regulatory costs of deploying monitoring networks, sampling inspection, and enforcement are high. The sustainability of regulatory input is in turn constrained by local performance assessment structures. The compliance improvements brought by campaign-style enforcement are difficult to maintain over the long run (43). The third is misaligned incentives and payoff spillovers. The incremental costs of plastic reduction. These include replacing buoys with environmentally friendly ones, recovering aging facilities, closed-loop management of operational waste, and third-party testing. They are borne by enterprises alone, whereas the health benefits of exposure reduction are shared non-excludably by all consumers. Although the consumption side exhibits significant willingness to pay for microplastic mitigation technologies in aquatic products (44). Whether this willingness can be converted into a premium that enterprises actually capture still depends on the existence of credible disclosure channels. The three barriers compound one another, meaning that unilateral government regulation is cost-constrained, spontaneous enterprise plastic reduction lacks recoverable incentives. Individual consumer protection can act only at the end of the chain. No unilateral strategy suffices to sustain source governance in the long run.

This predicament is precisely the situation characterized by polycentric governance theory. Effective governance of complex public affairs depends on the sustained interaction of multiple decision centers under rule constraints, beyond what a single government or a pure market can accomplish (45). Applied to the marine ranching setting, the governance system is composed of three types of actors coupled through the three channels of regulation, the market, and information. The government sits at the supply end of the regulatory channel. Its available instruments include binding arrangements such as material admission and end-of-life recovery standards for plastic facilities. Monitoring and sampling inspection cover aquaculture waters, sediments, organisms, and marketed products. It also imposes penalties and mandated rectification within a prescribed period for violators. Incentive arrangements such as retrofit subsidies and green certification for plastic-reducing enterprises. Informational instruments such as disclosure of test results, traceability labeling, risk communication, and reporting rewards. The regulatory leverage of informational instruments has empirical support. A causal study of U. S. occupational safety regulation showed that publicizing a single violation improved peer-firm compliance by roughly the equivalent of 210 additional on-site inspections (46). The EU Single-Use Plastics Directive brings plastic-containing fishing gear under extended producer responsibility. The continuing negotiation of a legally binding global plastics agreement indicates that source regulation of fishery and aquaculture plastics has entered the institutional agenda (31, 47). Marine ranching enterprises, as the direct point sources of aquaculture-derived microplastics. They determine the release intensity at the head of the chain by whether they implement plastic-reduction retrofits such as material substitution, facility inspection and recovery, closed-loop shore disposal of operational waste, and depuration. Consumers are both the ultimate bearers of exposure consequences and shapers of production-side behavior through two reverse channels. On the demand side, they convert willingness to pay into enterprises’ compliance premium through label recognition and green payment. On the oversight side, they raise the probability that violations are verified and exposed through complaints, reporting, and public opinion, constituting a social complement to limited enforcement resources. Integrating these actor roles and interaction channels yields the tripartite collaborative governance framework for dietary microplastic exposure risk in marine ranching shown in Figure 1. The governance strategies of the three actors are as follows.

(1) The government. Its strategy set is {strong regulation, weak regulation}. “Strong regulation” is not a single instrument but a package of regulatory arrangements: formulating and enforcing material admission, end-of-life recovery, and ledger management standards for marine ranch plastic facilities (buoys, netting, ropes, feed packaging). Establishing a microplastic monitoring and sampling inspection network covering aquaculture waters, sediments, organisms, and marketed products. Granting facility retrofit subsidies and green certification support to enterprises practicing plastic-reduction farming. Penalizing violators according to law and ordering rectification within a prescribed period. Disclosing test results, promoting traceability labels carrying microplastic risk information, and conducting public-facing dietary risk communication. “Weak regulation” means the government maintains only routine administration such as aquaculture sea-use licensing and production statistics, without investing dedicated monitoring, enforcement, or information disclosure resources.
(2) Marine ranching enterprises. Their strategy set is {plastic-reduction farming, conventional farming}. “Plastic-reduction farming” means the enterprise makes incremental investments to achieve whole-process source reduction: replacing weathering-prone expanded polystyrene (EPS) buoys with high-density polyethylene or polyurea-coated integrally molded buoys (48). Regularly inspecting and recovering aging netting, ropes, and raft accessories. Implementing closed-loop shore management of feed packaging and operational waste. Establishing third-party microplastic testing and product traceability systems. Applying depuration to marketed shellfish to lower residual particle loads. “Conventional farming” means continuing to use low-cost EPS buoys and polyethylene gear, abandoning damaged or expired facilities in place, and conducting no testing or traceability.
(3) Consumers. Their strategy set is {active participation, passive participation}. “Active participation” is a composite behavior of demand guidance and social oversight. It means actively recognizing traceability labels and test information, paying a premium for low-microplastic-risk products. It also means adopting consumption practices that reduce intake such as depuration treatment and removal of the digestive gland, and participating in oversight through channels such as complaints, reporting, and social media feedback. “Passive participation” means consumers base purchases only on price and sensory quality, without searching for risk information, paying a premium, or participating in oversight.

**Figure 1:**
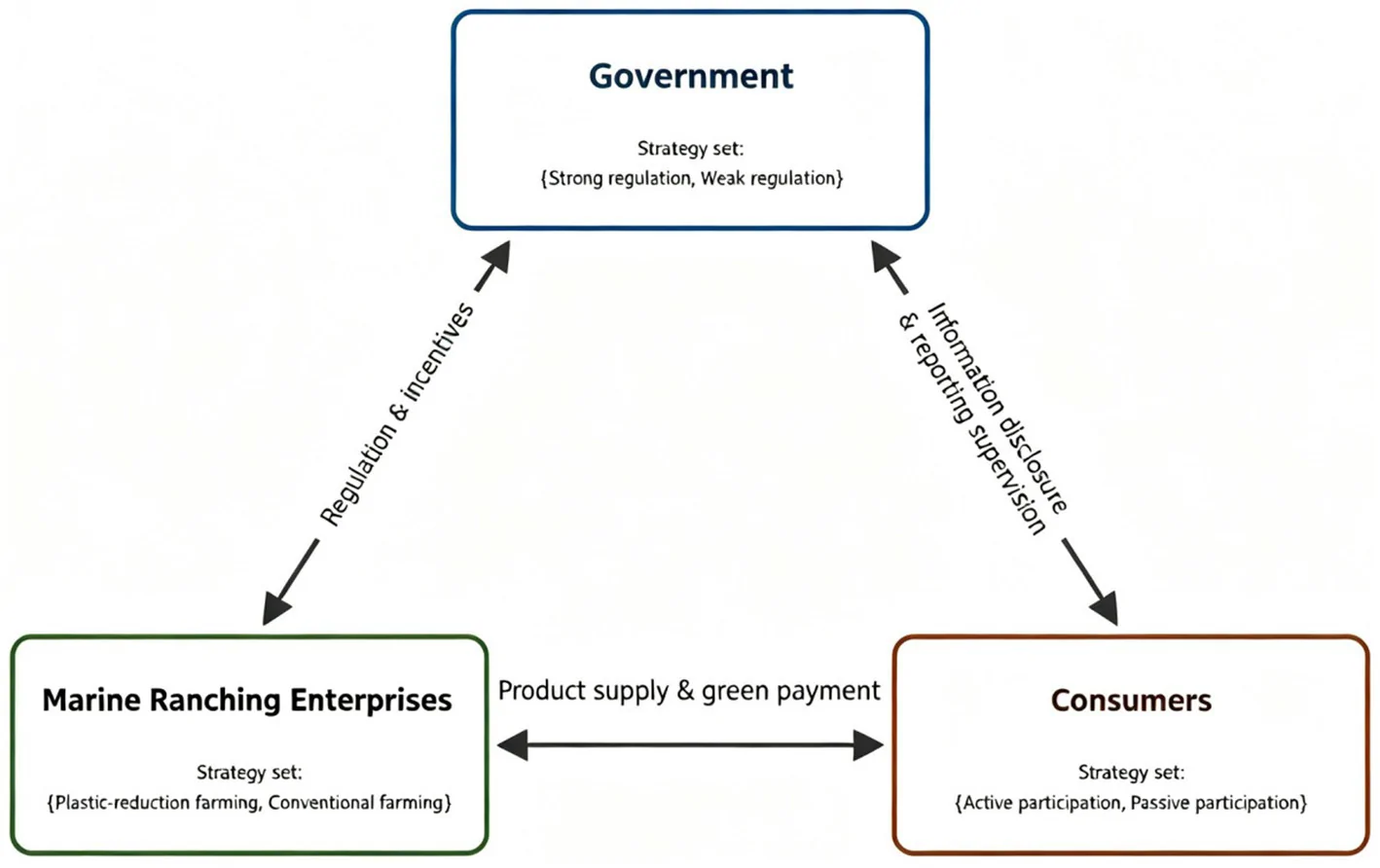
Tripartite collaborative governance framework for dietary microplastic exposure risk in marine ranching.

### 2.2 Construction of the evolutionary game model

The manifestation of dietary microplastic exposure risk in marine ranching involves significant time lags and causal ambiguity. Microplastic information is strongly asymmetric, and ordinary consumers cannot identify it through the senses. The production and disclosure of microplastic information is therefore itself a public good requiring government investment, calling for environmental regulation by the government. Meanwhile, operators’ decisions display pronounced peer effects. Marine ranching operators are numerous and geographically clustered. Information on the costs and benefits of plastic-reduction retrofits spreads horizontally mainly through industry associations and upstream–downstream supply chain channels. The profit and loss outcomes of a few first movers are observed and imitated by many followers. Taken together, the governance of microplastics in marine ranching is in essence the outcome of repeated trial and error, learning, and adjustment among the government, marine ranching enterprises, and consumers in long-term interaction. It is not a one-shot static choice. Evolutionary game theory is premised on bounded rationality, drives dynamic changes in strategy frequencies through fitness differences within populations, and characterizes the time path along which the system converges to an evolutionarily stable strategy. This matches closely the mechanism design needs of the collaborative microplastic governance examined here. Accordingly, this study constructs a tripartite evolutionary game model.

#### 2.2.1 Model assumptions

(1) Assumptions on government behavior. The government’s objective function comprises the social cost to public health and the payoff from governance performance. Let the probability that the government chooses strong regulation be *x*, and the probability that it chooses weak regulation be 1 − *x*, with *x*∈[0,1]. Let *Cg* denote the incremental regulatory cost of strong relative to weak regulation. It covers the deployment and operation of microplastic monitoring transects, sampling inspection of marketed shellfish, enforcement patrols, standard setting, construction of traceability and information disclosure platforms, and risk communication. Let *S* denote the government’s facility retrofit subsidy and green certification reward to plastic-reducing enterprises. Let *L* denote the portion of the social cost to public health induced by dietary microplastic exposure that is borne by the government, including medical security expenditure, monitoring and emergency expenditure, and regional industry reputation losses. The governance performance payoff must be divided into an outcome-oriented and a process-oriented component. The first is the outcome-based payoff *V*: when the region’s marine ranch products as a whole exhibit low microplastic risk, the government gains enhanced credibility and a regional public brand premium. This payoff has a public-good character, so even if the government chooses weak regulation while enterprises reduce plastics spontaneously, the government still shares *V*. The second is the process-based payoff *ΔV*. The current ecological environment and food safety assessment systems evaluate not only governance outcomes but also assign scores directly to institutional inputs such as monitoring system construction, enforcement performance, and information disclosure. The government therefore obtains *ΔV* as long as it implements strong regulation, regardless of enterprise compliance. Distinguishing the two is necessary. If only the outcome-based payoff *V* were specified, then once enterprises had generally reduced plastics the government’s net payoff from strong regulation would always be −(*Cg*+*S*). A fully collaborative governance configuration could then never be stable under any parameters, contradicting the observed fact that regulators remain continuously present. Conversely, let *P* denote the accountability loss the government bears if it chooses weak regulation and a risk event is exposed, including public opinion response, emergency handling, and accountability from higher levels. As for penalties and rectification, when an enterprise’s violation is verified. In addition to paying the fine *F*, it bears the combined loss *D* of reputational damage, order losses, and rectification within a prescribed period. It is also ordered to retrofit its facilities, which partially lowers the regional exposure level in the current period. Let *A* denote the reward the government grants for verified consumer reports.
(2) Assumptions on marine ranching enterprise behavior. An enterprise’s plastic-reduction farming implements whole-process source reduction through incremental investment. This includes replacing weathering-prone expanded polystyrene buoys with high-density polyethylene or polyurea-coated integrally molded buoys, regularly inspecting and recovering aging netting and ropes. It also covers closed-loop shore management of feed packaging and operational waste, establishes third-party testing and traceability systems, and applies depuration to marketed shellfish. Let the probability that the enterprise chooses plastic-reduction farming be *y*, and the probability that it chooses conventional farming be 1 − *y*, with *y*∈[0,1]. Let *π* denote the baseline net payoff of conventional farming, and let *Ce* denote the incremental cost of plastic-reduction relative to conventional farming. It covers the annualized expenditures on the facility replacement, recovery and disposal, testing and traceability, and depuration described above. We impose *Ce*>*S*, i.e., the government subsidy does not fully cover the incremental cost of plastic-reduction retrofits, to preclude the “subsidy fraud” and subsidy dependence induced by over-subsidization. Whether the incremental cost of plastic-reduction farming can be recovered depends on whether consumers can recognize it and are willing to pay for it. Let *R* denote the premium and market expansion payoff the enterprise obtains when consumers participate actively, i.e., the sum of the price premium of traceable low-microplastic-risk products over ordinary products and the expansion of market share. When consumers participate passively, the enterprise can still obtain a partial premium *βR* through non-retail channels such as export certification, chain supermarket admission, and government green procurement. Here β∈(0,1) is the premium discount coefficient that reflects the partial recognition of plastic-reduced products by non-retail channels. The enterprise-side premium payoff *R* is jointly determined by the consumer-side unit premium payment *p* and the expansion of the green market share, and the two move in the same direction. As for exposure reduction, let *γ* denote the proportion by which source plastic reduction lowers the dietary exposure level (0 < *γ* < 1). Its real-world content is the degree to which measures such as facility material substitution, recovery of aging facilities, and closed-loop management of operational waste cut microplastic release into the aquaculture waters. When an enterprise chooses conventional farming but is caught and ordered to rectify within a prescribed period, source reduction is partially realized in the current period, and the residual exposure proportion is written as (1 − *λγ*).
(3) Assumptions on consumer behavior. Whether the government implements information disclosure directly changes consumers’ information acquisition costs. Let the probability that consumers choose active participation be *z*, and the probability that they choose passive participation be 1 − *z*, with *z*∈[0,1]. Let *Cc* denote the basic cost of active consumer participation, covering information search and label recognition, adjustments to consumption practices such as depuration treatment, and the time and effort of complaints and reporting. Under strong regulation, consumers can rely on government-disclosed test information and traceability labels, so the participation cost is exactly *Cc*. Let *ΔCc* denote the additional information screening cost consumers bear under weak regulation owing to the lack of authoritative disclosure channels and unified labeling. Active consumer participation is a composite behavior of demand guidance and social oversight. On the oversight side, in addition to raising the detection probability from *λ*_2_ to *λ*_1_ as described above. Under weak regulation, active consumer oversight can also bring about media or public exposure with probability *μ*, creating a reputational deterrent against violating enterprises. On the demand side, let *p* denote the premium consumers pay for low-microplastic-risk products. Let *U*_0_ denote the baseline utility consumers obtain from consuming marine ranch aquatic products. Let *H* denote the health loss from dietary microplastic exposure borne by consumers under conventional farming with no consumption-side protection, expressed as a monetized health utility loss including risk-perception anxiety. As for exposure protection, let *σ* denote the proportion by which consumption-side protective behavior (choosing traceable products, depuration treatment, removing the digestive gland, etc.) lowers dietary exposure (0 < *σ* < 1). Because source reduction and consumption-side protection act on different links of the serial pathway “facility fragmentation and release → bioaccumulation → processing and consumption → human intake” and are mutually independent. The residual exposure proportion under their joint action is (1 − *γ*) (1 − *σ*), rather than a simple sum. It must be emphasized that plastic-reduction farming lowers the average microplastic load of the region’s marketed products, and its health benefit is shared by all consumers without excludability. Hence passively participating consumers, though paying no premium *p* and bearing no participation cost *Cc*, still obtain the exposure reduction of proportion *γ*, constituting typical free-riding behavior.

Based on the above assumptions, there are 8 possible strategy combinations of the government, marine ranching enterprises, and consumers. The payoff functions of the three actors under each combination are shown in Table 1.

**Table 1:** Payoff matrix of the government–marine ranching enterprise–consumer tripartite game.

Strategy combination	Government	Marine ranching enterprise	Consumer
Strong regulation, plastic-reduction farming, active participation	*V* + *ΔV* − *C_g_* − *S* − (1 − *γ*)*L*	*π* − *C_e_* + *S* + *R*	*U*_0_ − *p* − *C_c_* − (1 − *γ*) (1 − *σ*)*H*
Strong regulation, plastic-reduction farming, passive participation	*V* + *ΔV* − *C_g_* − *S* − (1 − *γ*)*L*	*π* − *C_e_* + *S* + *βR*	*U*_0_ − (1 − *γ*)*H*
Strong regulation, conventional farming, active participation	*ΔV* + *λ*_1_*F* − *λ*_1_*A* − *C_g_* − (1 − *λ*_1_*γ*)*L*	*π* − *λ*_1_(*F* + *D*)	*U*_0_ − *C_c_* + *λ*_1_*A* − (1 − *λ*_1_*γ*) (1 − *σ*)*H*
Strong regulation, conventional farming, passive participation	*ΔV* + *λ*_2_*F* − *C_g_* − (1 − *λ*_2_*γ*)*L*	*π* − *λ*_2_(*F* + *D*)	*U*_0_ − (1 − *λ*_2_*γ*)*H*
Weak regulation, plastic-reduction farming, active participation	*V* − (1 − *γ*)*L*	*π* − *C_e_* + *R*	*U*_0_ − *p* − *C_c_* − *ΔC_c_* − (1 − *γ*) (1 − *σ*)*H*
Weak regulation, plastic-reduction farming, passive participation	*V* − (1 − *γ*)*L*	*π* − *C_e_* + *βR*	*U*_0_ − (1 − *γ*) *H*
Weak regulation, conventional farming, active participation	−(1 − *μγ*)*L* − *μP*	*π* − *μD*	*U*_0_ − *C_c_* − *ΔC_c_* − (1 − *μγ*) (1 − *σ*) *H*
Weak regulation, conventional farming, passive participation	−*L*	*π*	*U*_0_ − *H*

#### 2.2.2 Model formulation

Based on the payoff matrix in Table 1, the expected payoffs and replicator dynamic equations of the three actors are derived in turn. First, the government’s replicator dynamic equation. Let the expected payoffs of the government choosing strong and weak regulation be *E*_*G*1_ (Equation 1) and *E*_*G*2_ (Equation 2) respectively, and let the population average expected payoff be *E_G_*. The replicator dynamic equation of the government’s strategy then follows, as shown in Equation 3.


$$\begin{array}{l} E_{G1}=\mathrm{\Delta V}+y[V-C_{g}-S-(1-\gamma )L]+(1-y)\\ \{\begin{array}{l} z[\lambda_{1}F-\lambda_{1}A-C_{g}-(1-\lambda_{1}\gamma )L]+(1-z)\\ \left[\lambda_{2}F-C_{g}-(1-\lambda_{2}\gamma )L\right] \end{array}\} \end{array}$$ (1)

$$E_{G2}=y[V-(1-\gamma )L]+(1-y)[z(-(1-\mu \gamma )L-\mathrm{\mu P})-(1-z)L]$$ (2)

$$\begin{array}{l} f_{1}(x,y,z)=x(1-x)(E_{G1}-E_{G2})=x(1-x)\\ \{\begin{array}{l} \mathrm{\Delta V}+(1-y)[\begin{array}{l} z(\begin{array}{l} \lambda_{1}F-\lambda_{1}A+\\ (\lambda_{1}-\mu )\mathrm{\gamma L}+\mathrm{\mu P} \end{array})+\\ (1-z)(\lambda_{2}F+\lambda_{2}\mathrm{\gamma L})-C_{g} \end{array}]-\\ y(C_{g}+S) \end{array}\} \end{array}$$ (3)

Second, the marine ranching enterprise’s replicator dynamic equation. Let the expected payoffs of the enterprise choosing plastic-reduction and conventional farming be (Equation 4) *E*_*E*1_ and *E*_*E*2_ (Equation 5) respectively, and let the population average expected payoff be *E_E_*. The replicator dynamic equation of the enterprise’s strategy then follows, as shown in Equation 6.


$$E_{E1}=\pi -C_{e}+\mathrm{xS}+[\beta +(1-\beta )z]R$$ (4)

$$E_{E2}=\pi -x[\lambda_{2}+(\lambda_{1}-\lambda_{2})z](F+D)-(1-x)\mathrm{z\mu D}$$ (5)

$$\begin{array}{l} f_{2}(x,,y,,z)=y(1-y)(E_{E1}-E_{E2})=y(1-y)\\ \{\begin{array}{l} \mathrm{xS}+[\beta +(1-\beta )z]R-C_{e}+\\ x[\lambda_{2}+(\lambda_{1}-\lambda_{2})z](F+D)+\\ (1-x)\mathrm{z\mu D} \end{array}\} \end{array}$$ (6)

Third, the consumer’s replicator dynamic equation. Let the expected payoffs of consumers choosing active and passive participation be (Equation 7) *E*_*C*1_ and *E*_*C*2_ (Equation 8) respectively, and let the population average expected payoff be *E_C_*. The replicator dynamic equation of the consumer’s strategy then follows, as shown in Equation 9.


$$\begin{array}{l} E_{C1}=x\{\begin{array}{l} y[U_{0}-p-C_{c}-(1-\gamma )(1-\sigma )H]+\\ \left(1-y)[\begin{array}{l} U_{0}-C_{c}+\lambda_{1}A-(1-\lambda_{1}\gamma )\\ (1-\sigma )H \end{array}\right] \end{array}\}+\\ (1-x)\{\begin{array}{l} y[\begin{array}{l} U_{0}-p-C_{c}-\mathrm{\Delta Cc}-\\ (1-\gamma )(1-\sigma )H \end{array}]+\\ \left(1-y)[\begin{array}{l} U_{0}-C_{c}-\mathrm{\Delta Cc}-\\ (1-\mu \gamma )(1-\sigma )H \end{array}\right] \end{array}\} \end{array}$$ (7)

$$\begin{array}{l} E_{C2}=x\{y[(1-\gamma )H]+(1-y)[U_{0}-(1-\lambda_{2}\gamma )H]\}+\\ (1-x)\{y[U_{0}-(1-\gamma )H]+(1-y)(U_{0}-H)\} \end{array}$$ (8)

$$\begin{array}{l} f_{3}(x,y,z)=z(1-z)(E_{C1}-E_{C2})=z(1-z)\\ \{\begin{array}{l} y[(1-\gamma )\mathrm{\sigma H}-p]-C_{c}-(1-x)\Delta C_{c}+\\ x(1-y)[(\sigma (1-\lambda_{1}\gamma )+(\lambda_{1}-\lambda_{2})\gamma )H+\lambda_{1}A]+\\ (1-x)(1-y)[\sigma (1-\mu \gamma )+\mu \gamma ]H \end{array}\} \end{array}$$ (9)

Fourth, the replicator dynamic system of the tripartite evolutionary game. Combining Equations 3, 6, 9 yields the three-dimensional replicator dynamic system characterizing the co-evolution of the strategies of the government, marine ranching enterprises, and consumers, as shown in Equation 10. It is a three-dimensional nonlinear autonomous differential dynamical system defined on the unit cube *Ω* = {(*x*,*y*,*z*)|0 ≤ *x*,*y*,*z* ≤ 1}. Its right-hand-side functions are continuously differentiable on *Ω*, so a solution exists and is unique, and all faces, edges, and vertices of *Ω* are invariant sets, i.e., system trajectories cannot escape *Ω*.


$$\begin{array}{l} \mathrm{dx}/\mathrm{dt}=x(1-x)\{\mathrm{\Delta V}+(1-y)[z(\lambda_{1}F-\lambda_{1}A+\\ (\lambda_{1}-\mu )\gamma L+\mu P)+(1-z)(\lambda_{2}F+\lambda_{2}\mathrm{\gamma L})-\\ \mathrm{Cg}]-y(\mathrm{Cg}+S)\}\\ \mathrm{dy}/\mathrm{dt}=y(1-y)\{\mathrm{xS}+[\beta +(1-\beta )z]R-C_{e}+\\ x[\lambda_{2}+(\lambda_{1}-\lambda_{2})z](F+D)+\\ (1-x)\mathrm{z\mu D}\}\;\\ \mathrm{dz}/\mathrm{dt}=z(1-z)\{y[(1-\gamma )\mathrm{\sigma H}-p]-C_{c}-\\ (1-x)\Delta \mathrm{Cc}+x(1-y)[(\sigma (1-\lambda_{1}\gamma )+\\ (\lambda_{1}-\lambda_{2})\gamma )H+\lambda_{1}A]+(1-x)\\ (1-y)[\sigma (1-\mu \gamma )+\mu \gamma ]H\} \end{array}$$ (10)

### 2.3 Simulation model and parameter calibration

The replicator dynamic system of the tripartite evolutionary game is a three-dimensional nonlinear differential dynamical system with no analytical solution except for a few special parameter combinations. Although the stability analysis has provided the necessary and sufficient conditions for each equilibrium, it cannot answer along what path and at what speed the system converges to a stable state. Moreover, the stability conditions reflect only qualitative parameter relationships and cannot indicate which parameters have the most critical marginal influence within realistic value ranges. This study therefore further solves the replicator dynamic system numerically. The simulation uses the fourth-order Runge–Kutta method with integration step *h* = 0.002. The simulation horizon is set to 30 to 400 evolutionary time units according to scenario needs, and after each iteration the solution is truncated within *Ω* to ensure *x*, *y*, *z* ∈ [0,1]. The program is implemented in Python 3. Evolutionary time *t* is a dimensionless game-round scale whose conversion to natural time depends on the actual frequency with which the three actors adjust their strategies.

(1) Production and operation parameters (*π*, *C_e_*, *R*, *β*, *D*) come from field surveys of and interviews with operators in national marine ranching demonstration zones. Output and value data are drawn from in fishery statistical yearbooks and market quotations for environmentally friendly aquaculture facilities. (2) Regulatory parameters (*C_g_*, *S*, *F*, *A*, *V*, *ΔV*, *L*, *P*, *λ*_1_, *λ*_2_, *μ*) come from the dedicated budgets, enforcement ledgers, and sampling inspection records of local marine fishery and market regulation departments. They also draw on current provisions of ecological environment target responsibility assessment measures and food safety reward-for-reporting measures. Among them, *L* is estimated from the disease burden and medical expenditure attributable to dietary microplastic exposure, and *P* from the emergency response costs of comparable food safety incidents. (3) Among the health and consumption parameters (*H*, *γ*, *σ*, *p*, *C_c_*, *ΔC_c_*), *H* is converted from dietary microplastic exposure assessments of marine ranch aquatic products combined with willingness-to-pay or monetized disease burden methods. *γ* refers to the share of aquaculture sources in the regional microplastic flux and the abatement efficiency of facility substitution. *σ* refers to the reduction rates of microplastic residues in bivalves achieved in depuration experiments and by digestive gland removal. *p*, *C_c_*, and *ΔC_c_* come from consumer questionnaire surveys. The specific parameter values are given in Table 2.

**Table 2:** Baseline parameter values for the simulation model.

Parameter	Baseline value	Calibration basis and notes
*π*	10.0	Nondimensionalization benchmark, representing the annual net payoff of conventional farming for a unit operator
*C_e_*	2.0	Annualized incremental cost of replacing buoys and gear with environmentally friendly ones, recovering aging facilities, closed-loop management of packaging and operational waste, and building third-party testing and traceability systems
*R*	2.5	Sum of the price premium of traceable low microplastic risk products over ordinary products and the expansion of market share
*β*	0.35	Under passive consumer participation, recognition comes only from export certification, chain supermarket admission, and government green procurement channels
*D*	3.5	Sum of reputational loss, order losses, and rectification expenditure within a prescribed period, notably higher than the fine
*F*	1.0	Penalty magnitude under current marine environmental protection and aquatic product quality and safety rules
*S*	1.0	Facility retrofit subsidy, covering roughly half of the incremental plastic-reduction cost
*C_g_*	1.2	Expenditure on monitoring transect operation, market sampling inspection, enforcement patrols, and the information disclosure platform, apportioned to a unit operator
*ΔV*	2.4	Process-based assessment payoff
*V*	3.0	Outcome-based performance payoff, which can also be shared under weak regulation
*L*	4.5	Social cost to public health borne by the government
*P*	3.0	Accountability loss after a risk event is exposed under weak regulation
*A*	0.15	Reward-for-reporting standard
*λ*_1_	0.65	Probability that an enterprise violation is verified when consumers participate actively
*λ*_2_	0.30	Probability that an enterprise violation is verified when consumers participate passively
*μ*	0.15	Probability that a violation is exposed by the media or the public under weak regulation
*H*	5.0	Health loss from dietary exposure borne by consumers under conventional farming with no consumption-side protection
*γ*	0.55	Reduction rate of the dietary exposure level achieved by source plastic reduction
*σ*	0.30	Reduction rate of the dietary exposure level achieved by consumption-side protective behavior
*p*	0.4	Premium consumers pay for low microplastic risk products
*C_c_*	0.5	Basic cost of active consumer participation
*ΔC_c_*	0.7	Additional information screening cost under weak regulation
*U*_0_	12.0	Baseline consumption utility

The replicator dynamic equations depend only on the payoff differential between the two strategies within the same population and are invariant to positive linear transformations of each actor’s payoff function. Hence the absolute dimensions of the three parties’ parameters do not affect the evolutionary results. What matters is the relative magnitude of parameters within the same actor, and that parameters appearing simultaneously in two actors’ payoff functions (*S*, *F*, *A*, *D*) use the same accounting basis. Accordingly, this study takes the annual accounts of a unit marine ranching operator as the benchmark and nondimensionalizes all monetized parameters.

## 3 Results and analysis

### 3.1 Results and analysis of the evolutionary game model

Setting *f*_1_ = *f*_2_ = *f*_3_ = 0 in the replicator dynamic system yields all of its equilibrium points. Besides the 8 vertices of the unit cube *Ω*, the system has several mixed-strategy equilibria on the edges, faces, and interior of *Ω*. However, by the basic result for multi-population evolutionary games, an asymptotically stable equilibrium must be a strict Nash equilibrium, and a strict Nash equilibrium must be a pure-strategy equilibrium. Hence no mixed-strategy equilibrium can be an evolutionarily stable strategy, and it suffices to examine the 8 vertices of *Ω*, denoted in turn *E*_1_ (0,0,0), *E*_2_ (1,0,0), *E*_3_ (0,1,0), *E*_4_ (0,0,1), *E*_5_ (1,1,0), *E*_6_ (1,0,1), *E*_7_ (0,1,1), and *E*_8_ (1,1,1). By Lyapunov’s first method, an equilibrium is asymptotically stable when all three eigenvalues at that point are negative. When at least one eigenvalue is positive, the point is a saddle or an unstable node. When a zero eigenvalue appears while the others are non-positive, the case is critical and requires the center manifold theorem for further determination, which this study does not pursue under the assumption that parameters are in general position. Substituting the coordinates of the 8 vertices into the Jacobian matrix yields the eigenvalues at each equilibrium. Requiring all three eigenvalues to be negative gives the asymptotic stability condition of each equilibrium, as shown in Table 3.

**Table 3:** Asymptotic stability conditions of the equilibrium points.

Equilibrium	Real-world scenario	Population residual exposure	Asymptotic stability condition
*E*_1_ (0,0,0)	Complete governance failure	*H*	*ΔV* + *a*_2_ < *C_g_*; *C_e_* > *βR*; *c*_2_ < *C_c_* + *ΔC_c_*
*E*_2_ (1,0,0)	Regulator present, deterrence ineffective	(1 − *λ*_2_*γ*) *H*	*ΔV* + *a*_2_ > *C_g_*; *C_e_* > *S* + *βR* + *λ*_2_ (*F* + *D*); *c*_1_ < *C_c_*
*E*_3_ (0,1,0)	Market-driven voluntary plastic reduction	(1 − *γ*) *H*	*ΔV* < *C_g_* + *S*; *C_e_* < *βR*; (1 − *γ*) *σH* < *p* + *C_c_* + *ΔC_c_*
*E*_4_ (0,0,1)	Consumers participating alone	(1 − *μγ*) (1 − *σ*) *H*	*ΔV* + *a*_1_ < *C_g_*; *C_e_* > *R* + *μD*; *c*_2_ > *C_c_* + *ΔC_c_*
*E*_5_ (1,1,0)	Government–enterprise dyadic governance	(1 − *γ*) *H*	*ΔV* > *C_g_* + *S*; *C_e_* < *S* + *βR* + *λ*_2_ (*F* + *D*); (1 − *γ*)*σH* < *p* + *C_c_*
*E*_6_ (1,0,1)	High pressure, low efficiency	(1 − *λ*_1_*γ*) (1 − *σ*)*H*	*ΔV* + *a*_1_ > *C_g_*; *C_e_* > *S* + *R* + *λ*_1_ (*F* + *D*); *c*_1_ > *C_c_*
*E*_7_ (0,1,1)	Self-organized social co-governance	(1 − *γ*) (1 − *σ*)*H*	*ΔV* < *C_g_* + *S*; *C_e_* < *R* + *μD*; (1 − *γ*) *σH* > *p* + *C_c_* + *ΔC_c_*
*E*_8_ (1,1,1)	Full collaborative co-governance	(1 − *γ*) (1 − *σ*)*H*	*ΔV* > *C_g_* + *S*; *C_e_* < *S* + *R* + *λ*_1_ (*F* + *D*); (1 − *γ*) *σH* > *p* + *C_c_*

Population residual exposure is the dietary microplastic exposure loss borne by consumers in the corresponding equilibrium state, benchmarked against the exposure loss H under conventional farming with no consumption-side protection.

As Table 3 shows, the 8 equilibria fall into three tiers by public health consequence: *E*_7_ and *E*_8_ achieve source reduction and consumption-side protection simultaneously, with the lowest population residual exposure, and are the target states of governance. *E*_3_ and *E*_5_ achieve source reduction only, with the next-lowest exposure. *E*_2_, *E*_4_, and *E*_6_ rely on enforcement detection or consumption-side protection acting alone, with limited reduction. *E*_1_ is the worst state, with full exposure. Comparing the stability conditions of the points further yields the following five findings.

(1) The watershed between government presence and exit. A necessary condition for the asymptotic stability of *E*_5_ and *E*_8_ is *ΔV* > *C_g_* + *S*, whereas a necessary condition for *E*_3_ and *E*_7_ is *ΔV* < *C_g_* + *S*. The two are mutually exclusive. This shows that once the enterprise population has generally shifted to plastic-reduction farming, whether the government maintains strong regulation depends entirely on whether the process-based assessment payoff *ΔV* covers the net regulatory cost *Cg + S.* It is independent of the outcome-based payoff *V*. This result reveals a structural contradiction of collaborative governance: outcome-oriented performance incentives cannot support sustained regulation, because governance success itself can be shared by free-riding. Only direct assessment of process inputs such as monitoring system construction, enforcement performance, and information disclosure can lock in the government’s presence. Correspondingly there are three policy paths. The first is incorporating microplastic monitoring capacity and information disclosure into the ecological environment assessment indicator system. The second is substituting online monitoring, remote sensing identification, and third-party testing for manual patrols. The third is designing a subsidy phase-down mechanism that declines as the enterprise plastic-reduction share rises.
(2) The tiered threshold structure of enterprise plastic reduction. The tolerance ceilings of the four plastic-reduction equilibria for the incremental retrofit cost *C_e_* are, in turn, *βR* (*E*_3_), *R* + *μD* (*E*_7_), *S* + *βR* + *λ*_2_(*F* + *D*) (*E*_5_), and *S* + *R* + *λ*_1_(*F* + *D*) (E8). They are ordered as *βR* < *R* + *μD* < *S* + *R* + *λ*_1_(*F* + *D*) and *βR* < *S* + *βR* + *λ*_2_(*F* + *D*) < *S* + *R* + *λ*_1_(*F* + *D*). That is, the threshold of *E*_3_ is the lowest and that of *E*_8_ the highest, while the relative ranking of *E*_5_ and *E*_7_ depends on the relative strength of fiscal incentives and the market premium. This tiered structure shows that when all three parties are in place. The triple stacking of the fiscal incentive *S*, the full green premium *R*, and the punishment deterrent under the highest detection probability *λ*_1_(*F* + *D*) gives enterprises the highest bearable ceiling for plastic-reduction costs. Whereas in *E*_7_, with the government absent, the market premium *R* and the exposure deterrent *μD* must support the retrofit cost on their own.
(3) The self-erosion effect of consumer participation. The stability of both *E*_7_ and *E*_8_ requires (1 − *γ*)*σH* > *p* + *C_c_* (*E*_7_ must additionally cover *ΔC_c_*). The left-hand side declines monotonically as the source reduction rate γ rises. The more successful enterprise plastic reduction is, the lower the residual exposure, and the smaller the marginal benefit to consumers of continuing to pay the premium and invest in oversight. This constitutes a negative feedback in which governance success erodes participation incentives. Consumption-side participation does not automatically consolidate with governance success but may instead disintegrate because of it, degrading *E*_8_ into *E*_5_ and *E*_7_ into *E*_3_. It must be stressed that the feasible way to sustain consumer participation is not to heighten risk perception but to press down the cost side of participation. The first is lowering Cc through unified traceability labeling and disclosure of test information. The second is lowering the premium p through the scaled-up production of green aquatic products.
(4) The leverage value of information disclosure. Comparing the consumer conditions of *E*_8_ and *E*_7_ shows that they differ only by the term *ΔC_c_*: when the government implements strong regulation, the threshold for consumers to maintain active participation is lower by *ΔC_c_* than when the government is absent. The same directional relaxation can be observed when enterprises do not reduce plastics. The condition for active consumer participation relaxes from *c*_2_ > *C_c_* + *ΔC_c_* under weak regulation (*E*_4_) to *c*_1_ > *C_c_* under strong regulation (*E*_6_)_._ This incorporates both the elimination of *ΔCc* and the payoff increment brought by raising the detection probability from *λ*_2_ to *λ*_1_. This means that information disclosure unilaterally relaxes the consumer participation constraint in all configurations, and this relaxation relies on no fiscal expenditure.
(5) Multiple steady states, path dependence, and persistent oscillation. A compatibility check of the stability conditions of any two equilibria shows that, under the model’s parameter constraints (0 < *μ* < *λ*_2_ < *λ*_1_ ≤ 1, *C_e_* > *S*, *A* < *F*), only the pairs (*E*_1_, *E*_6_) and (*E*_2_, *E*_4_) can hold simultaneously. The stability conditions of any other two points are mutually exclusive. The latter pair requires *a*_1_ < *a*_2_, i.e., the reporting reward expenditure induced by consumer oversight exceeds the payoff from improved detection efficacy, which exists only in an extremely narrow parameter interval. Hence the only multiplicity of practical relevance is (*E*_1_, *E*_6_): both belong to the case where enterprises have not reduced plastics, differing only in whether the government and consumers are activated. This indicates that path dependence occurs only in the stage before source reduction has started, when the initial regulatory intensity and consumer risk awareness determine whether the system falls into “complete failure” or “high pressure, low efficiency.” Once the enterprise population shifts to plastic-reduction farming, the system’s long-run state is uniquely determined by the parameters.
(6) Governance cycles exist under specific conditions. All eight sets of conditions may fail simultaneously. In that case no asymptotically stable pure-strategy equilibrium exists in *Ω*, and the three parties’ strategies exhibit persistent periodic oscillation. It corresponds to the real-world governance cycle of “risk event exposure → regulatory tightening → enterprise rectification → regulatory slackening → risk resurgence.” For public health, this state is inferior to any stable equilibrium in which source reduction has been achieved. The population’s dietary exposure level repeatedly rebounds with the regulatory cycle and the long-term cumulative dose is markedly higher than under stable governance.

### 3.2 Numerical simulation results and analysis

This study designs three groups of scenarios to test the government, consumer, and enterprise channels, respectively. Each scenario varies a single parameter while all others stay at baseline, so as to isolate the net effect of a single policy instrument. All simulations start from the initial state (0.5, 0.5, 0.5). To translate strategy evolution into public health consequences, a population-average residual dietary exposure index is defined: Ẽ(*t*) = [1 − *γ*(*y* + (1 − *y*)*λ̄*)](1 − *σz*), *λ̄* = *x*[*λ*_2_ + (*λ*_1_ − *λ*_2_)*z*] + (1 − *x*)*μz*. The first bracket captures source reduction: the share *y* of enterprises practicing plastic-reduction farming cuts exposure by *γ*, and the portion of the remaining enterprises that is caught and ordered to rectify (expected share *λ̄*) achieves the same cut. The second bracket captures consumption-side protection: the share *z* of consumers reduces their own exposure by *σ* through choosing traceable products and adjusting consumption practices.

#### 3.2.1 Scenario 1: duty-performance assessment incentives and the government’s sustained presence

When *ΔV* = 1.5 < *C_g_* + *S* = 2.2 (Figure 2a), the system has no evolutionarily stable strategy, and the government and enterprise strategies enter large periodic oscillations with a period of about 29 evolutionary time units. The mechanism is clearly discernible: the government withdraws from strong regulation because the assessment payoff cannot cover the net regulatory cost, whereupon enterprises abandon plastic reduction after losing subsidies and punishment deterrence. Widespread violations in turn raise the government’s net enforcement payoff and the payoff from avoiding accountability, prompting it to re-enter. Once enterprises are forced to rectify, strong regulation again becomes a pure cost burden, and the cycle restarts. Consumers become briefly active during each episode of enterprise violation and fall back rapidly as enterprises rectify.

**Figure 2:**
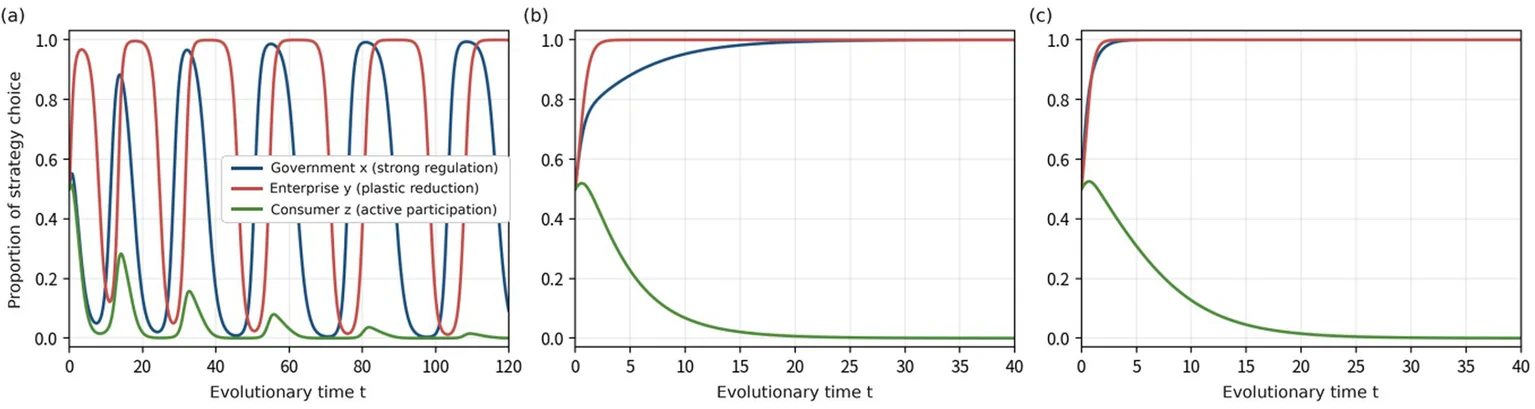
Strategy evolution paths under varying duty-performance assessment incentive. **(a)** ΔV = 1.5 < C_g_ + S = 2.2. **(b)** ΔV = 2.4 (baseline). **(c)** ΔV = 3.5.

In Figures 2b,c, the system converges to *E*_5_ (1,1,0) in both cases, with the same long-run residual exposure of 0.450. The difference lies in the convergence structure: with *ΔV* = 3.5 the government’s strategy reaches *x* > 0.99 within about 2.9 time units, versus 17.9 in the baseline. Yet the system’s overall convergence time increases slightly from 18.2 to 21.9 time units. This seemingly paradoxical result stems from a secondary effect: the government’s faster arrival eliminates the additional information screening cost *ΔC_c_* of weak regulation, which correspondingly slows consumers’ exit from active participation. It corroborates, from the side, the supporting role of information disclosure for consumption-side participation, but also shows that strengthening assessment and accountability alone cannot solve the absence of the consumption side.

#### 3.2.2 Scenario 2: consumption-side participation costs and governance regime transition

In Figure 3, the government and enterprise curves of the three cases almost coincide, and the differences appear entirely in the consumer dimension, which itself shows that information-type policy instruments act with clear specificity. When *C_c_* = 0.30 (Figure 3b), *p* + *C_c_* = 0.70 still slightly exceeds the threshold (1 − *γ*) *σH* = 0.675, so *z* still tends to 0 eventually, but the decay is extremely slow. At t = 40, *z* is still as high as 0.170. At t = 100 it is 0.044, and the system’s convergence time lengthening from 18.2 in the baseline to 160.3 time units. This is the classic phenomenon of critical slowing down. As parameters approach a bifurcation threshold, the driving force of the replicator dynamics tends to zero and the system lingers near a saddle point for a long time. Its practical implication deserves the attention of monitoring authorities: judging from a few years of observational data that consumer participation is “rising steadily” may well misread a transition process that will ultimately fall back. In Figure 3c, *p* + *C_c_* < 0.675, the consumer participation condition is satisfied, *z* rises monotonically and converges to 1, the system jumps to *E*_8_ (1,1,1), and the long-run residual exposure index falls from 0.450 to 0.315. It must be stressed that this improvement mobilizes no additional fiscal resources. The fall in Cc comes from the standardization of test result disclosure and traceability labeling, whose cost is mainly a one-off platform construction outlay. Once produced, the test information can be disclosed to all consumers at near-zero marginal cost.

**Figure 3:**
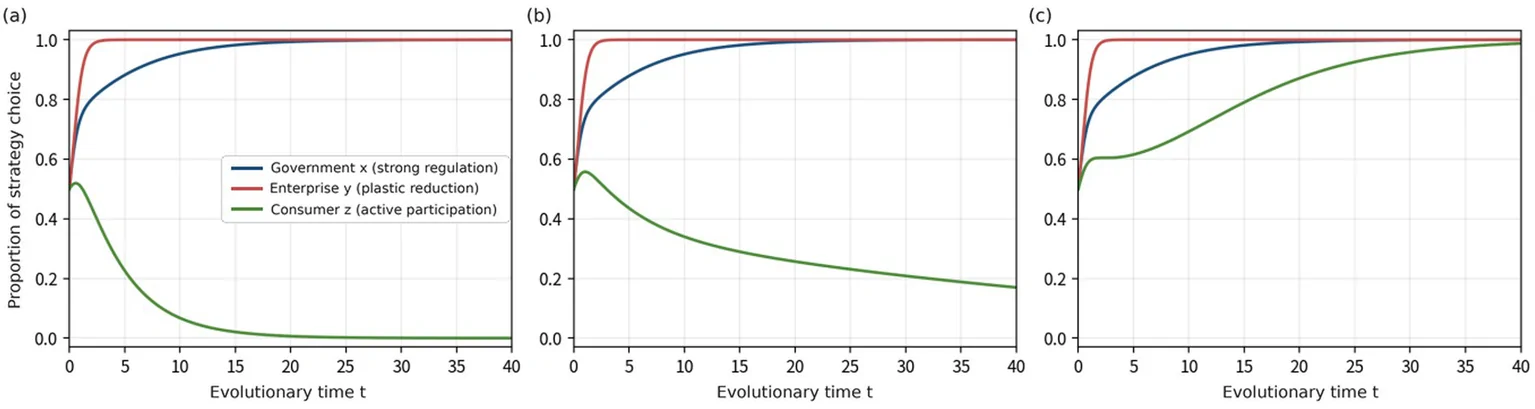
Strategy evolution paths under varying consumer participation cost. **(a)** C_C_ =0.50 (baseline). **(b)** C_C_ = 0.30. **(c)** C_C_ = 0.15.

#### 3.2.3 Scenario 3: rising compliance costs and two types of governance failure

At the baseline *C_e_* = 2.0 (Figure 4a), enterprises steadily choose plastic-reduction farming and the system converges to *E*_5_. *C_e_* = 4.5 (Figure 4b) lies between the two thresholds: above *S* + *βR* + *λ*_2_ (*F* + *D*) = 3.225 and below *S* + *R* + *λ*_1_ (*F* + *D*) = 6.425. The system again loses its evolutionarily stable strategy, but the oscillation pattern differs sharply from Scenario 1: the government maintains strong regulation throughout, and the oscillation occurs between enterprises and consumers. Enterprises briefly reduce plastics under subsidies and deterrence, then fall back under cost pressure. Consumers become highly active during episodes of enterprise violation (*z* peaks at 0.8) and recede after enterprises rectify. The period is about 26 time units, and the residual exposure index oscillates within [0.350, 0.626]. The average exposure of this oscillatory state is slightly below the baseline’s 0.450. This does not mean that rising compliance costs are beneficial. Rather, periodic enterprise violations repeatedly activate consumers, whose self-protection partially offsets the interruption of source reduction. But the dispersion of exposure levels in the oscillatory state is markedly larger. The repeated rectification by enterprises and the government’s sustained high-intensity regulation both incur adjustment costs far above the steady state. This makes it undesirable on both the public health and the economic efficiency dimensions.

**Figure 4:**
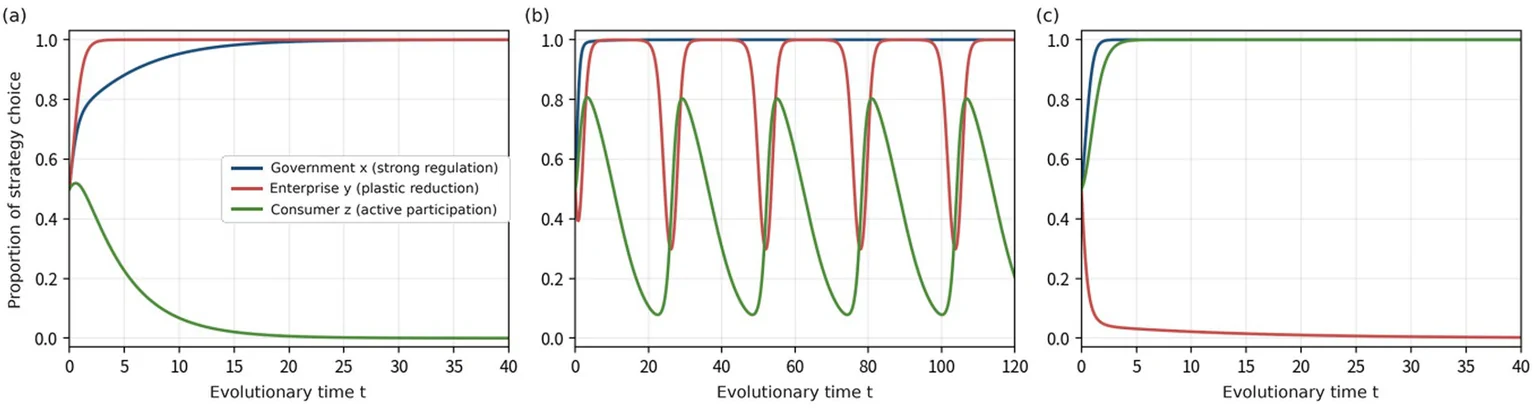
Strategy evolution paths under varying incremental plastic-reduction cost. **(a)** C_e_ =2.0 (baseline). **(b)** C_e_ = 4.5. **(c)** C_e_ = 6.5.

Once *C_e_* = 6.5 (Figure 4c) exceeds the upper threshold, the enterprise strategy converges rapidly to conventional farming while the government and consumers both maintain high input. The system then locks into the “high pressure, low efficiency” configuration *E*_6_ (1,0,1). The long-run residual exposure index is then 0.450, almost identical to the baseline’s *E*_5_, but the path to it is entirely different. E5 relies on source reduction, whereas E6 relies on the stacking of enforcement detection and consumption-side protection, with both the government and consumers in a state of sustained high-cost input. The contrast makes clear that when compliance costs are too high, intensifying regulation and mobilizing consumer participation can only maintain, not improve, health outcomes. The real way out is to lower Ce itself, by pressing down enterprises’ compliance threshold through the scale-up of environmentally friendly materials, technical support for facility retrofits, and dedicated financing arrangements.

#### 3.2.4 Comparing the effectiveness of policy instruments

The three scenario groups above each vary a single parameter and are not yet sufficient for comparing the relative effectiveness of different policy instruments. To this end, centered on the baseline values, six classes of policy-relevant parameters are varied individually over the interval of 0.25 to 2.5 times, and the long-run residual exposure index and convergence time are reported. The results are shown in Figure 5.

**Figure 5:**
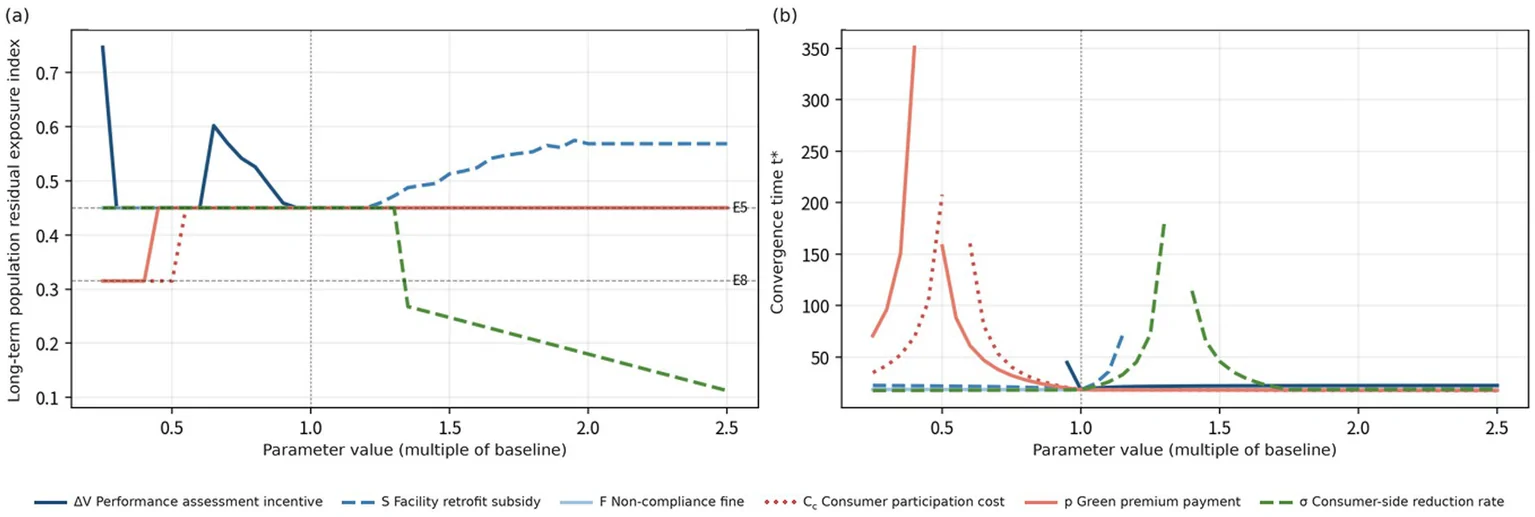
Dose–response relationships of six policy parameters. **(a)** Long-term residual exposure level. **(b)** Convergence speed (breaks indicate non-convergence).

The first class is fiscal and enforcement instruments. The non-compliance fine *F* has no effect whatsoever on long-run exposure over the entire interval examined (the constant 0.450 horizontal line in Figure 5a). Under the baseline parameters the enterprise plastic-reduction stability condition *C_e_* < *S* + *βR* + *λ*_2_ (*F* + *D*) is satisfied with a wide margin. Marginal changes in the fine alter the stability of no equilibrium. The duty-performance assessment incentive *ΔV* matters only when it falls below *C_g_* + *S*, and its effect is to destabilize rather than improve. Throughout that interval the system has no evolutionarily stable strategy, and long-run residual exposure rises to 0.45–0.75, varying irregularly with the oscillation pattern. Most noteworthy is the facility retrofit subsidy *S*. Once *S* exceeds *ΔV* − *C_g_* = 1.2 (i.e., 1.2 times the baseline), the net regulatory cost *C_g_* + *S* overtakes the assessment payoff *ΔV*. The government exits strong regulation, the system loses its stable equilibrium, and long-run residual exposure rises from 0.450 to about 0.57. This means the subsidy has a ceiling determined by the government’s own incentive constraint.

The second class is consumption-side instruments. The consumer participation cost *C_c_*, the green premium payment *p*, and the consumption-side reduction rate *σ* all have clear transition thresholds: when *C_c_* < 0.275, *p* < 0.175, or *σ* > 0.400, the system jumps from *E*_5_ to *E*_8_ and long-run residual exposure falls to 0.315. Among the three, *σ* is the most effective and does not saturate after the transition reaching 0.113 at *σ* = 0.75, only a quarter of the baseline level. This suggests that raising the uptake and effectiveness of consumption-side practices such as depuration and digestive gland removal may be the intervention path with the highest health return per unit input within this modeling framework. Figure 5b shows that pronounced spikes in convergence time appear near all thresholds. This regularity has a direct implication for policy evaluation: when a measure brings the system close to but not across a threshold. If the policy is withdrawn at that point because progress seems slow, the system will quickly fall back to its original state. The observation window of policy evaluation must therefore be longer than the time scale of critical slowing down.

## 4 Discussion

(1) The subsidy ceiling: a failure mechanism that should be located on the government side. That green subsidies have a ceiling is a consensus, but existing explanations concentrate on the enterprise side, and has a threshold-type negative effect on the total factor productivity of recipient enterprises. A recent tripartite evolutionary game study of marine ranching enterprises, the government, and consumers has also noted the following pattern. When the subsidy scale is too large, the government exits cooperation under the fiscal burden and the system loses its evolutionarily stable strategy (40, 49). This study’s conclusion points in the same direction but pushes the mechanism one step further. The subsidy does not work through the broad channel of “fiscal burden” but appears directly on the right-hand side of the stability condition *ΔV* > *C_g_* + *S* for the government to maintain strong regulation. Its threshold *ΔV − Cg* can be given analytically and read directly from assessment system parameters. At the same time, the outcome-based payoff is completely eliminated in the payoff differential between strong and weak regulation, showing that what the subsidy crowds out is not “governance enthusiasm” but a presence incentive unrelated to governance outcomes. The two explanations therefore imply different policy prescriptions. The enterprise-side explanation points to “linking subsidies to performance.” The government-side explanation points to “jointly determining subsidy scale with the scoring intensity of duty-performance assessment.” The same subsidy level is safe under an assessment system with a high *ΔV* and harmful under one with a low *ΔV*.
(2) Consumers are neither a demand parameter nor an unconditional improvement term. In tripartite evolutionary game studies of marine ranching and marine ecological governance, the third party is mostly set as a higher-level government, a local government, or a third-party testing institution (39, 50, 51). Consumers, where they appear, are usually treated as an exogenous demand or price variable. Yet empirical research shows that consumers hold measurable and heterogeneous willingness to pay for microplastic mitigation technologies in aquatic products (44), so exogenizing this dimension does not fit the facts. After making consumers an independent strategic actor, this study identifies three coupled channels: information disclosure, social oversight, and market demand. These cannot arise in dyadic or “government–enterprise–third party” structures, and their quantified consequences are considerable under the baseline calibration. But the tripartite structure also exposes a negative case invisible to existing frameworks. In E6 (1,0,1), the government and consumers both invest heavily. The residual exposure (1 − *λ_1_γ*) (1 − *σ*)*H* = 0.450 is exactly the same as in *E_5_* with only government–enterprise dyadic governance. Total social cost is far higher. Treating “public participation” as an unconditionally positive variable is not robust. Consumption-side mobilization is a conditional complement.
(3) A purely incentive-structural explanation of “campaign-style governance.” The periodic tightening and slackening in environmental regulation is usually attributed to the intermittence of campaign-style enforcement in resource mobilization and authority redistribution. This makes lasting constraints hard to form (43). Scenario 1 offers a complementary explanation that does not rely on those conditions. As long as the assessment system does not score process inputs separately, the outcome-based payoff is completely eliminated in the payoff differential between strong and weak regulation. Strong regulation is not evolutionarily stable near the compliance equilibrium. In other words, even if enforcement capacity and fiscal resources remain unchanged and no central–local information asymmetry exists, the relapse will still occur.

Research limitations and future directions. First, actor homogeneity. The model does not distinguish large enterprises from small household farms in retrofit costs and financing constraints, nor consumers of different income and risk-perception levels in willingness to pay. Since the public health significance of dietary exposure risk concentrates on high-intake and high-sensitivity subgroups, the homogeneity assumption may understate exposure inequality within the population. Multi-population evolutionary games or network games are natural extensions. Second, spatial and transboundary attributes. This study examines a closed governance system within a specific sea area and the portion of microplastics attributable to marine ranching aquaculture sources. It does not model land-based runoff inputs, shipping activities, or cross-area transport, nor horizontal games among local governments or the vertical coupling of watersheds and sea areas.

## 5 Conclusions and implications

### 5.1 Conclusion

Addressing the governance predicament of dietary microplastic exposure risk in marine ranching under climate change, in which release on the production side is misaligned with the burden borne on the consumption side. This study constructed a tripartite evolutionary game model of the government, marine ranching enterprises, and consumers. It analyzed the asymptotic stability conditions of all pure-strategy equilibria and used numerical simulation to systematically examine how each actor’s behavioral strategies affect the system’s convergence path, convergence speed, and long-term residual exposure. The results were then read against the climate risk and health expenditure nexus, in which climate change amplifies the source hazard and raises the health expenditure at stake for the government, enterprises, and households. The conclusions are as follows.

(1) Whether the government can sustain regulation depends solely on whether the process-based assessment payoff covers the net cost of regulation. The outcome-based performance payoff is completely canceled out in the payoff differential because it can be free-ridden, and once subsidies exceed the government-side ceiling they crowd out regulation and cause exposure to rebound. Subsidy phase-down is not only needed to guard against enterprise moral hazard but is a precondition for maintaining the government’s regulatory incentive.
(2) Enterprises’ tolerance ceiling for plastic-reduction retrofit costs follows a tiered threshold structure. It is highest when all three parties are in place and sharply tightened when the government is absent. The simultaneous phase-down of subsidies and regulation readily triggers a governance relapse.
(3) Consumer participation is subject to a self-undermining mechanism whereby governance success erodes participation incentives. The more successful enterprise plastic reduction is, the lower the residual exposure in the water and the products, the smaller the marginal health benefit to consumers of continuing to pay the green premium and invest in oversight. The target equilibrium may therefore degrade into a state supported only by the government and enterprises.
(4) The marginal effectiveness of different policy instruments differs markedly, and the system exhibits critical slowing down near thresholds. Information disclosure is the most cost-effective of all the policy instruments. First, it eliminates the additional information cost consumers would incur to assess microplastic content themselves. Second, it raises the expected payoff of active participation by increasing the probability that violations are verified. When the eight sets of equilibrium conditions all fail simultaneously, the system has no evolutionarily stable strategy and the three parties’ strategies exhibit persistent periodic oscillation. It corresponds to the real-world governance cycle of “risk event exposure → regulatory tightening → enterprise rectification → regulatory slackening → risk resurgence.”

### 5.2 Policy recommendations

(1) Improve the incentive and constraint mechanisms of government regulation. Incorporate process-based indicators into local ecological environment protection and aquatic product quality and safety assessment systems, raising the duty-performance assessment payoff so that it stably covers the net cost of regulation. Set the subsidy scale reasonably and establish a phase-down mechanism linked to assessment intensity. Subsidy scale should be jointly determined with the intensity of process-based assessment. This ensures that the post-subsidy net regulatory cost does not exceed the duty-performance assessment payoff, thereby preventing subsidies from exceeding the government-side ceiling at the source. Establish a subsidy phase-down mechanism that declines gradually as the enterprise plastic-reduction share rises. Tie subsidy disbursement to verified plastic-reduction performance. Checkable indicators such as the replacement rate of environmentally friendly buoys, the recovery rate of aging facilities, and the closed-loop shore disposal rate of operational waste serve as disbursement conditions to improve subsidy targeting.
(2) Establish a microplastic test information disclosure and aquatic product traceability labeling system. First, build a microplastic monitoring network covering aquaculture waters, sediments, organisms, and marketed products, and standardize the procedures of sampling, digestion, separation, and polymer identification. Publish test results to the public periodically, changing the information asymmetry created by microplastic content as a credence attribute. Second, implement a traceability labeling regime for the microplastic load of marine ranch products. A unified label is affixed to products that pass plastic-reduction certification and meet low-load standards. Consumers can then distinguish plastic-reduced from conventional products at low recognition cost, and green willingness to pay is converted into a market premium enterprises actually capture.
(3) Cultivate the green aquatic product premium mechanism and open up consumption-side participation channels. First, relying on traceability labels and authoritative test information, establish a green aquatic product certification system and organize branded marketing and consumer guidance. Plastic-reduced products then earn a stable quality premium, and consumers’ willingness to pay for low-microplastic aquatic products becomes an increment of revenue enterprises can anticipate. Second, encourage large-scale, standardized green aquaculture, lowering the unit premium through the scaled-up production of green aquatic products so that more consumers can afford the green payment and the market capacity of green demand expands.
(4) Establish monitoring and early warning for periodic oscillation scenarios. When monitoring data show alternating rises and falls in the enterprise plastic-reduction share, regulatory intensity, and consumer participation. This should be identified as a governance cycle with no stable equilibrium rather than a transitional state. The cycle of “exposure → tightening → rectification → slackening → resurgence” should be interrupted promptly through information disclosure and strengthened assessment. This prevents the population’s dietary exposure from repeatedly rebounding with the regulatory cycle.

## Data Availability

The original contributions presented in the study are included in the article/supplementary material, further inquiries can be directed to the corresponding author.
